# Piplartine Analogues and Cytotoxic Evaluation against Glioblastoma

**DOI:** 10.3390/molecules23061382

**Published:** 2018-06-08

**Authors:** Flávio Rogério da Nóbrega, Ozlem Ozdemir, Sheila Cristina S. Nascimento Sousa, Joice Nascimento Barboza, Hasan Turkez, Damião Pergentino de Sousa

**Affiliations:** 1 Laboratory of Pharmaceutical Chemistry, Universidade Federal da Paraíba, João Pessoa 58051-085, Brazil; frnobrega@hotmail.com (F.R.d.N.); joice_nascimentob@hotmail.com (J.N.B.); 2 Department of Molecular Biology and Genetics, Erzurum Technical University, Erzurum 25240, Turkey; ozlem.ozdemir@erzurum.edu.tr (O.O.); hasanturkez@gmail.com (H.T.); 3 Programa de Pós-Graduação em Engenharia Química, Universidade Federal de Sergipe, São Cristóvão 49100-000, Brazil; shcrisnascimento@yahoo.com.br; 4 Department of Pharmacy, “G. d’Annunzio” University of Chieti-Pescara, Via dei Vestini 31, 66013 Chieti Scalo, CH, Italy

**Keywords:** cancer, cytotoxic activity, antitumor, phenylpropanoid, alkaloid, natural product, esters, oxidative stress, *Piper*, synthetic derivatives

## Abstract

Piplartine (**1**) is an alkamide extracted from plants of the genus *Piper* which shows several pharmacological properties, including antitumor activity. To improve this activity, a series of analogues based on **1** have been synthesized by esterification and amidation using the 3,4,5-trimethoxycinnamic acid-like starting material. During the study, the moieties 3-(3,4,5-trimethoxyphenyl)acrylate and 3-(3,4,5-trimethoxyphenyl)acrylamide were maintained on esters and amides respectively. Meanwhile, functional changes were exploited, and it was revealed that the presence of two aromatic rings in the side-chain was important to improve the cytotoxic activity against the U87MG cell line, such as the compound (*E*)-benzhydryl 3-(3,4,5-trimethoxyphenyl)acrylate (**10**), an ester that exhibited strong cytotoxicity and a similar level of potency to that of paclitaxel, a positive control. Compound **10** had a marked concentration-dependent inhibitory effect on the viability of the U87MG cell line with apoptotic and oxidative processes, showing good potential for altering main molecular pathways to prevent tumor development. Moreover, it has strong bioavailability with non-genotoxic and non-cytotoxic properties on human blood cells. In conclusion, the findings of the present study demonstrated that compound **10** is a promising agent that may find applications combatting diseases associated with oxidative stress and as a prototype for the development of novel drugs used in the treatment of glioblastoma.

## 1. Introduction

Glioblastoma (GBM) is the most fatal primary brain cancer found in humans, with about 3 in 100,000 people developing the disease every year [1,2]. It originates from glial cells, but rarely disseminates outside the nervous system and grows diffusely into surrounding brain tissue. This cancer is still incurable and is characterized by nuclear atypia, mitotic activity, intense microvascular proliferation and necrosis. It occurs more frequently in males and there is no association with smoking [3], consumption of cured meat [4] or electromagnetic fields [5], however some viruses such as SV40 and HHV have a positive association [6,7]. Currently treatment for the cancer involves a multimodal approach which include surgery (total removal of tumor), post-operative radiation therapy radiotherapy (RT) and concurrent and adjuvant temozolomide (TMZ). The median survival rate is only 14.6 months with less than 3% to 5% of sufferers surviving longer than five years. Without treatment, the patient does not survive for more than 3 months [2].

The gliomagenesis occurs with a multistep process that exhibits complex patterns of abnormal gene expression [8]. The primary GBMs exhibit epidermal growth factor receptor (EGFR) amplification, phosphatase and tensin homolog (PTEN) mutation as well as the loss of chromosome 10. The secondary GBMs exhibit p53 mutation. And, glioblastoma tumor growth has been found as suppressed via *AKT*-mediated inhibition [9]. The ligation of EGFR by epidermal growth factor (EGF) induces endogenous generation of intracellular ROS and H_2_O_2_ in the cancer cell line. It is also clear that *PIP3K/AKT* pathways play an important role in the regulation of metabolism, apoptosis and survival in normal cells [10].

Piplartine (**1**) is an alkamide commonly found in long pepper (*Piper longum* L.). Previous reports indicated piplartine exhibited a wide range of pharmacological activities, including anti-diabetic, anti-ulcer, anti-platelet aggregation, anti-atherosclerotic, anti-depressant, anti-bacterial and anti-fungal properties [11,12,13,14]. Moreover, this compound possesses a highly selective and effective antitumor property, which protects against various types of tumors [15,16]. It has recently been demonstrated that piplartine kills HGG (high-grade glioma) cells by PRDX4 inactivation inducing endoplasmic reticulum stress [10]. Therefore, the antitumor activity of piplartine (**1**) in a type of cancer of the Central Nervous System motivated us to investigate a collection of synthetic analogues of piplartine on human U87MG glioblastoma cell line, as well as exploring the molecular genetic basis underlying their effects for the first time.

## 2. Results

### Chemistry

**Figure 1:**
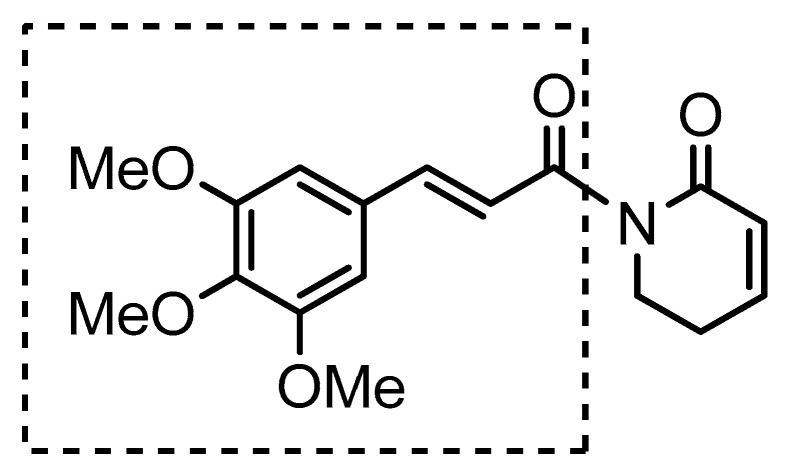
Chemical structure of piplartine (**1**).

**Scheme 1:**
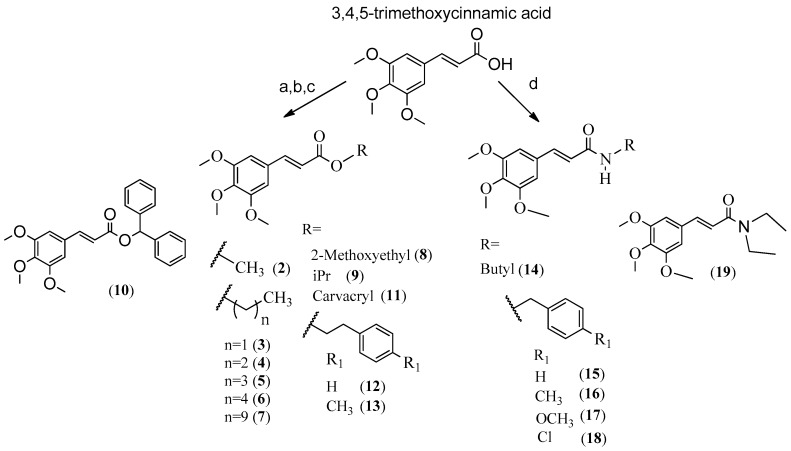
Reagents and conditions: (a) ROH, H_2_SO_4_, reflux; (b) Et_3_N, RBr or RCl, acetone, reflux; (c) SOCl_2_, reflux, ROH (d) RNH_2_ or (CH_3_CH_2_)_2_NH, BOP, Et_3_N, CH_2_Cl_2_, DMF.

For this study, 18 analogues of **1** (Figure 1) were synthesized, maintaining the similar moiety (*E*)-3-(3,4,5-trimethoxyphenyl)acryloyl in the esters and amides (Scheme 1). Side-chain modifications were also evaluated by changing the radical (R) to: methyl (**2**), ethyl (**3**), propyl (**4**), butyl (**5**), pentyl (**6**), decyl (**7**), 2-methoxyethyl (**8**), iPr (**9**), CHPh_2_ (**10**), carvacryl (**11**), phenethyl (**12**), 4-methylphenethyl (**13**) on esters and butyl (**14**), benzyl (**15**), 4-methylbenzyl (**16**), 4-methoxylbenzyl (**17**), 4-chlorobenzyl (**18**) and *N*,*N*-diethyl (**19**) on amides. Side-chain modifications are expected to increase lipophilicity and antitumor efficacy of the esters and amides, as well as their mode of interaction with the receptor (Scheme 1) [17,18,19].

To synthesize the compounds, it starting materials were utilized like 3,4,5-trimethoxycinnamic acid, ROH to esters and primary or secondary amines to amides. The reactions used were (a), (b), (c) to synthesize esters and (d) to synthesize amides (Scheme 1). Reactions were performed in one step and most products were obtained in high yield.

**Figure 2:**
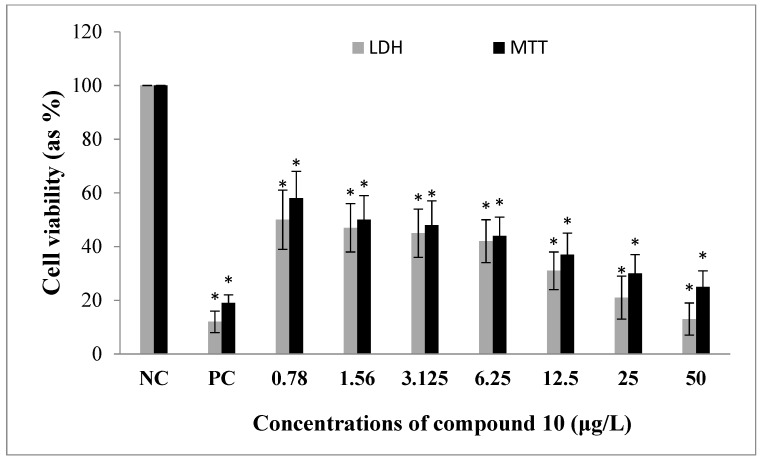
The in vitro effects of compound **10** on the cell viability of human U87MG glioblastoma cell line. Data are presented as the mean ± S.D. of four independent experiments. NC: Negative control, PC: Positive control (as paclitaxel). * *p* < 0.05, comparison to the NC.

To evaluate the cytotoxic potential of compounds **2**–**19** against human U87MG, glioblastoma cells were used with several concentrations (0 to 50 μg/mL). After 48 h of incubation, cell viability rates were established by MTT and LDH analysis. IC_50_ values of each analogue were determined due to observed cell viability results after MTT assay. All tested analogues led to inhibition of cellular proliferation rates as compared to untreated cells in a clear concentration and compound type dependent manners, as illustrated in Table 1 and Figure 2. In this sense, the most potent cytotoxic agent among those tested was the compound **10** against U87MG cells.

**Figure 3:**
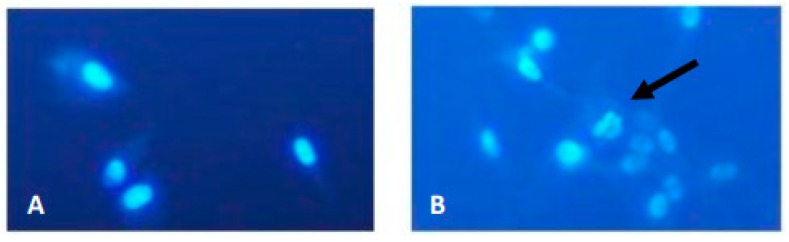
Hoechst 33,258 staining in U87MG cells. (**A**) Negative control; (**B**) Cells treated with compound **10** for 48 h at IC_50_ concentration (arrow shows nuclear fragmentation, magnification ×400).

Cell apoptosis induced by compound **10** was examined by Hoechst 33,258 staining and observed under a fluorescence microscope at 400× magnification. U87MG cells were treated with compound **10** for 48 h at their IC_50_ concentrations. The nuclear fragmentation of treated cells was compared with the control group and characteristic alterations of apoptosis including enhanced chromatin condensation and nuclear fragmentation were detected in the compound **10** treated cells (Figure 3).

**Figure 4:**
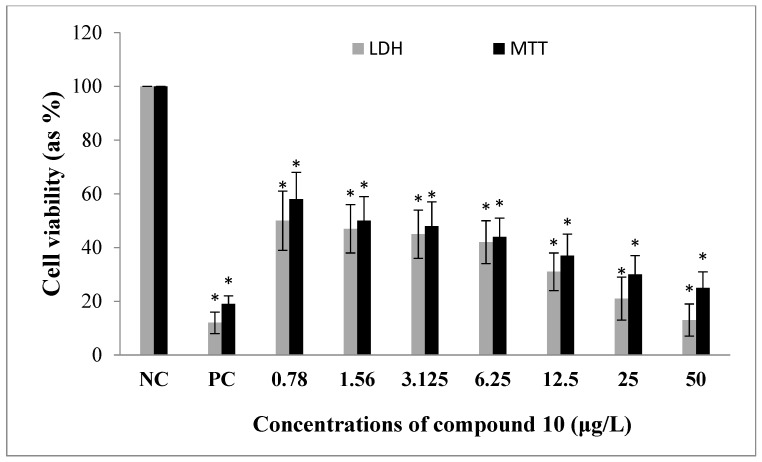
The effects of compound **10** on total antioxidant capacity (TAC) levels in cultured U87MG cells for 48 h. Data are expressed as the mean ± S.D. of four independent experiments. NC: Negative control, PC: Positive control (as ascorbic acid, 10 μM). * *p* < 0.05, comparison to NC.

The total antioxidant capacity (TAC) and the total oxidant status (TOS) levels were evaluated in samples from treated and untreated cultures using colorimetric measurement methods. As shown from the results presented in Figure 4, concentrations (0.78 and 50 μg/mL) of compound **10** resulted in a significant increase of TAC levels on U87MG cells compared with the controls. On the other hand, treatments with compound **10** did not change the TOS levels in cultured U87MG cells for all the concentrations (data not shown).

**Figure 5:**
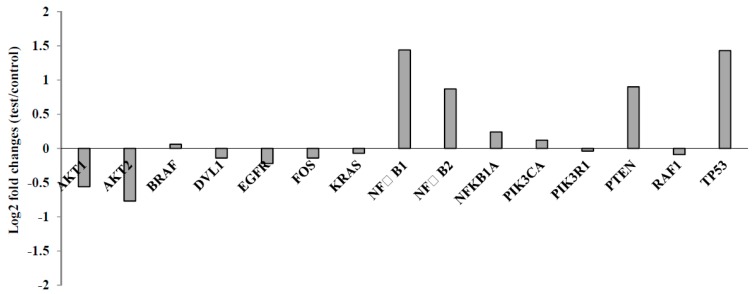
Gene expression alterations after treatment with compound **10** on U87MG cells for 48 h.

To find out the differences in the level of gene expression in U87MG cells exposed using compound **10**, a custom plate with 15 different genes involved in cancer related pathways was used via qRT-PCR studies. According to the gene expression results, two genes (*AKT1* and *AKT2*) were significantly (>0.5 fold change) down-regulated and four genes (*NF-ĸB1*, *NF-ĸB2*, *PTEN* and *TP53*) were up-regulated in the U87MG cells as compared to untreated subjects upon exposure to **10**. However, there were slight and insignificant alterations (<0.5 fold change) of nine genes, including BRAF, DVL1, EGFR, FOS, KRAS, NF-ĸB1A, RAF1, PIK3CA and *PIK3R1* (Figure 5).

To evaluate biosafety of **10**, its cytotoxic and genotoxic potential were investigated on cultured peripheral human whole blood (PHWB) cells. Firstly, the cytotoxicity testing was performed by MTT and LDH assays. The human blood cells exposed to 0.78 to 50 μg/mL of **10**. The results of both assays showed that **10** did not lead to any significant (*p* > 0.05) changes in cell proliferation. Afterwards, genotoxic potential was assessed by SCE testing and determination of 8-OH-dG. There were not significant differences in the frequencies of SCEs between the control group and **10** treated groups for 72 h (*p* > 0.05). Likewise, 8-OH-dG levels were not increased in the blood cells that were treated with **10** concentrations (Data not shown). Collectively, the **10** exhibited non-cytotoxic and non-genotoxic properties in PHWB cell cultures.

## 3. Discussion

### 3.1. Structure Activity Relationship (SAR)

Table 1 shows that the introduction of methylene groups into the radicals attached to the (*E*)-3-(3,4,5-trimethoxyphenyl)acrylate in compounds **2**–**6** provided IC_50_ values inversely proportional to the carbon chain size, with the exception of **3** (IC_50_: 100.030 ± 1.12 μg/mL). Comparing ester **5** (IC_50_: 57.871 ± 0.71 μg/mL) with amide **14** (IC_50_: 13.196 ± 0.32 μg/mL), it was observed that the butyl radical attached to the group 3,4,5-trimethoxycinnamoyl on amide might more effective than the same radical attached to the group (*E*)-3-(3,4,5-trimethoxyphenyl)-acrylate on esters. The introduction of methyl groups contributed to increase lipophilicity, but did not show an increase of cytotoxic activity when the compound **12** (IC_50_: 24.786 ± 0.37) was compared with **13** (IC_50_: 30.336 ± 0.35) [20].

For research carried out by Example [21], piplartine (**1**) and other compounds were isolated from the *Piper cernuum* plant and the pharmacological activity was made in several tumor cell lines. The cell lines evaluated by the MTT test were: BF16F10-Nex2 (murine melanoma), U87MG (human glioblastoma), HeLa (human cervical carcinoma), HCT (human colon carcinoma), HL-60 (human leukemic cell) and A2258 (human melanoma). The same author showed that **1** did not obtain significant results against these cancer cell lines (IC_50_: >200 μg/mL in all tested strains), but that one of the analogues, known as piplaroxide, did obtain moderate activity in the BF16F10-Nex2, HeLa and HL-60 strains. Piplaroxide differs from **1** by replacing the double bond between the 5′ and 6′ carbons by an epoxide and absence of the methoxyl at the 3-position of the ring.

The difference in chemical structure of **1** and piplaroxide is the oxygen atoms in the portion of the epoxide at the 5′ and 6′ carbons on piplaroxide. This oxygen may have contributed to increased piplaroxide antitumor activity as compared to **1** [21,22].

Comparing the side-chains R of compound **8** (IC_50_: 50.077 ± 0.62 μg/mL) and **4** (IC_50_: 78.393 ± 0.69 μg/mL), it was found that oxygen is also determinant to increase antitumor activity in the U87MG cell line. In accordance with the current research, the oxygen at the moieties provides a new center for H-bond that could influence the binding of the analogue to its target site [20]. However, the presence of the oxygen on methoxyl moiety on the *para* position on R_1_ of amide **17** (IC_50_: 22.654 ± 0.39) did not contribute to improve cytotoxic activity when compared to amide **16** (IC_50_: 22.741 ± 0.34), with the presence of the methyl group in this position of the ring.

In general, the presence of aromatic rings on side-chain R of the esters and amides increased antitumor activities against U87MG, but the trisubstituted aromatic ring present in the carvacryl moiety of ester **12** did not provide more effectiveness in comparison to the other compounds with aromatic rings on R.

The branch present on side-chains R of ester **9** (IC_50_: 81.433 ± 0.55) did not increase cytotoxic activity compared with other compounds with side-chains R composed with saturated radicals. However, the change of methyl groups to phenyl on branch radical of ester **10** (CI_50_: 2.579 ± 0.35) provide an IC_50_ more closely to a positive control paclitaxel (IC_50_: 2.119 ± 0.35) and also appeared to be the most active synthesized compound against the U87MG cell proliferation. Therefore, further experiments were performed with this compound.

### 3.2. Biological Activity

In cell biology, U87MG—an abbreviation for “Uppsala 87 Malignant Glioma”—is a primary lineage of glioblastoma, which, immortalized, originated from a 44-year-old cancer patient. This cell line is usually used to evaluate cytotoxic and genotoxic aspects of drugs and other molecules in brain cancer cells, especially glioblastomas [23]. Present data revealed that tested analogues exhibited anti-proliferative activity. Our findings also showed that compound **10** induced apoptosis in U87MG cells via leading increases of chromatin condensation and nuclear fragmentation. There is scarce data on the anti-glioblastoma actions of **2**–**19**. However, the cytotoxic activities of **1** are associated with the generation of ROS, and previous studies have demonstrated that **1** exhibited antiproliferative action via leading decreases of the expression of some key proteins (HER, JAK 1 and 2, STAT3, c-MET, GSTP1, JNK) that are responsible for the development of various cancer types [24,25,26]. Again, in a current investigation, it was determined that **1** served as a microtubule-destabilising agent in MCF-7 breast cancer cells [27]. And, the apoptotic action by **1** was associated to caspase-3-mediated poly (ADP-ribose) polymerase (PARP) cleavage and cell cycle arrest at G2/M phase in cultured human PC-3 prostate cancer cells [28].

Previous research revealed that **1** caused increases of intracellular level of reactive oxidative species (ROS) and inhibited tumor growth [11]. Disparately, present results indicated that oxidative alterations are not closely related to antitumor action by the novel and effective analogue, **10**. In supporting our novel finding on in vitro oxidative effects of **10** analogue, it was determined that most glioblastomas overexpressed ROS-degrading enzyme peroxiredoxin 4 (PRDX4) and suggested that pharmaceutical PRDX4 inactivation effectively kills the cancer cells by increasing ROS levels [10]. Therefore, it can be concluded that analogue **10** induced oxidative stress that was restored by overexpressed PRDX4 in cultured U87MG cells. Due to this, **1** was found to be less effective in SF-295 human glioblastoma cells as compared to HCT-8 human colon carcinoma cells [29].

Our results firstly revealed that **10** led to down-regulation of *AKT1* and *AKT2* genes in U87MG cells. Therefore, it was determined that the anticancer potential of **10** was largely associated with the PI3K/AKT/mTOR pathway, since AKT was an important mediator of this intracellular signaling pathway which is important in cell cycle regulation [30]. PI3K/AKT/mTOR was reported to play key functions on tumor initiation and progression as well as angiogenesis. AKT2 was found to be especially necessary for glioma cell migration [31]. In fact, the enhanced expression of AKT1 and cyclin D1 were detected in cultured U87MG cells [32]. Likewise, AKT1 and AKT2 found to be related to bcatenin/Tcf-4 signaling pathway that promoted malignant transformation in human LN229 glioma cell line. Moreover, AKT1 was considered to be more effective than AKT2 in malignant transformation. Thus, AKT1 and AKT2 were considered as valuable targets for glioblastoma therapy as well as a rational strategy for restraining the metastasis of gliomas [33]. Our positive control agent paclitaxel was known to down-regulate the MMP-9-mediated p38/JNK signaling pathway and block the proliferation of U87MG cells [34]. However, it was determined that the combined use of paclitaxel with alkylating agents supported a greater clinical efficacy of paclitaxel against malignant brain tumors [35]. But the adverse effects of these combinations are still perturbative. At this point, present findings show that **10** as anti-tumoral agent or suppressor with a good biosafety level is interrelated to proliferation and invasion of glioblastoma cells by inhibiting AKT1 signaling and **10** may be considered as an effective therapeutic treatment against glioblastoma.

The present qRT-PCR analysis revealed up-regulation of the genes involving NF-ĸB1, NF-ĸB2, PTEN and TP53 in vitro. Conversely, constitutive activation of NF-κB supported growth and survival of glioblastoma cells in human and mouse models [36]. However, it was determined that varied levels of NF-κB activation effected cancer propagation via several mechanisms. Thereof, it was considered that the alterations of the NF-κB signalling response in both upstream stimuli and the downstream targets would be fundamental for gliomagenesis in the near future [37]. Similarly, the overexpression of p53 and mutation in TP53 were associated with adverse outcomes in studies of malignant gliomas from children [38]. On the other hand, the tumor suppressor gene *PTEN* was shown to regulate Akt signalling, cellular growth and apoptosis [39]. Again, blocking the PTEN expression was linked to metastasis in addition to both radio- and chemotherapy responses in patents with glioma and breast cancer. Accordingly, PTEN is addressed as a main regulator of tumor sensitivity towards different therapeutic options [40,41,42,43,44,45,46,47]. In a recent study [47], it was revealed that PTEN was not the primary target of paclitaxel resistance and its regulator was evaluated as a substantive target for suppression of miR-22, cyclin B1 or combining with inhibitor of Akt, providing a successful strategy for paclitaxel therapy. In this regard **10** provides a rationale strategy against glioblastoma in single or combined use with other antineoplastic agents. Supplementary materials are available on line.

The human lymphocytes were suggested as a serviceable experimental model for planning chemotherapeutic strategies as well as dose selection from the point of genotoxic and cytotoxic damage potentials of drug/drug candidates on healthy cells [48,49]. Consequently, cultured peripheral human whole blood cells were used to evaluate the cytotoxic (MTT and LDH assays) and genotoxic (SCE and 8-OH-dG assays) potential of **10**. Finally, the property of **10** as a non-cytotoxic and non-genotoxic agent with high anti-cancer efficacy makes it a potential candidate for novel cancer therapy.

## 4. Materials and Methods

The ^1^H and ^13^C NMR, and IR signals assigned to analogues of **1** were comparing with signals already published. The unpublished molecules such as **6**, **7**, **8**, **10**, **13** and **16** besides the same spectrophotometric methods used to other analogues, was used LS-MALDI TOF/TOF to confirm the synthesis.

All reagents were purchased from Aldrich and were of commercial grade. IR spectra were recorded on Irprestige-21 Shimadzu Fourier Transform spectrophotometer (Shimadzu, Kyoto, Japan). ^1^H and ^13^C NMR spectra were recorded on a Varian Mercury spectrometer 200 MHz (^1^H) and 50 MHz (^13^C), Varian (Palo Alto, CA, USA). Chemical shifts are reported relative to the solvent peak CDCl_3_ or TMS. To HMRS was used the equipment ultraflex II MALDI TOF/TOF equipped with a Laser with High Performance (λ = 355 nm) and reflector operating by software FlexControl 2,4 (Bruker Daltonics G, bsH, Bremen, Germany) Melting points were measured on an equipment Tecnal PFM-II, 220 V.

### 4.1. General Procedure for Synthesis of Compounds ***2**–**5**, **8*** and ***9***

To a solution of 3,4,5-trimethoxycinnamic acid (0.25 g) in 250 mL of ROH, 0.5 mL 96% (*v*/*v*) H_2_SO_4_ was added under stirring. The reaction mixture was refluxed for 3 h. Half of ROH was removed under reduced pressure and the following solution was then diluted with 10 mL water and the product extracted with ethyl acetate. The organic phase of each reaction, when gathered were successively washed with 5% (*w*/*v*) NaHCO_3_ and water, dried over anhydrous Na_2_SO_4_ and filtered. After removal of ethyl acetate under vacuum, the pure products of each of those reactions were obtained [50].

#### 4.1.1. (*E*)-Methyl-3-(3,4,5-trimethoxyphenyl)acrylate (**2**)

Yield 93%; white solid, m.p.: 135–136 °C; MM: 252.10 g/mol; ^1^H NMR (200 MHz, CDCl_3_) δ_H_ 7.59 (d, *J* = 16.0 Hz, 1H), 6.73 (s, 2H), 6.33 (d, *J* = 16.0 Hz, 1H), 3.87 (s, 9H), 3.79 (s, 3H). ^13^C NMR (50 MHz, CDCl_3_) δ_C_ 167.5, 153.5, 144.9, 140.1, 129.9, 117.1, 105.2, 61.1, 56.2, 51.8, IR ν_max_ (KBr, cm^−1^) 3003, 1697, 2943, 2837, 1632, 1040, 1005, 1582, 1506, 851 [51,52].

#### 4.1.2. (*E*)-Ethyl-3-(3,4,5-trimethoxyphenyl)acrylate (**3**)

Yield 98.8%; white solid, MM: 266.12 g/mol, m.p.: 65–66 °C; ^1^H NMR (200 MHz, CDCl_3_) δ_H_ 7.53 (d, *J* = 16.0 Hz, 1H), 6.68 (s, 2H), 6.28 (d, *J* = 16.0 Hz, 1H), 4.19 (q, *J* = 6.0 Hz, 2H), 3.81 (s, 9H), 1.27 (t, *J* = 6.0 Hz, 3H), ^13^C NMR (50 MHz, CDCl_3_) δ_C_ 166.8, 153.3, 144.5, 139.9, 129.8, 117.4, 105.1, 60.8, 60.4, 56.0, 14.3, IR ν_max_ (KBr, cm^−1^) 2999, 2974, 2945, 2837, 1701, 1634, 1038, 997, 1583, 1506, 872 [52].

#### 4.1.3. (*E*)-Propyl-3-(3,4,5-trimethoxyphenyl)acrylate (**4**)

Yield 93%, beige solid, MM: 280.13 g/mol, m.p.: 97–100 °C; ^1^H NMR (200 MHz, CDCl_3_) δ_H_ 7.58 (d, *J* = 16.0 Hz, 1H), 6.74 (s, 2H), 6.34 (d, *J* = 16.0 Hz, 1H), 4.15 (t, *J* = 6.0 Hz, 2H), 3.87 (s, 9H), 1.72 (dt, *J* = 6.0, 8.0 Hz, 2H), 0.98 (t, *J* = 8.0 Hz, 3H), ^13^C NMR (50 MHz, CDCl_3_) δ_C_ 167.1, 153.5, 144.6, 140.0, 130.0, 117.6, 105.2, 66.2, 61.0, 56.2, 22.2, 10.6, IR ν_max_ (KBr, cm^−1^) 3003, 2941, 2841, 1697, 1632, 1005, 1585, 1506, 835 [53].

#### 4.1.4. (*E*)-Butyl-3-(3,4,5-trimethoxyphenyl)acrylate (**5**)

Yield 99.6%, brown solid, MM: 294.15 g/mol, m.p.: 74–75 °C; ^1^H NMR (200 MHz, CDCl_3_) δ_H_ 7.57 (d, *J* = 16.0 Hz, 1H), 6.73 (s, 2H), 6.33 (d, *J* = 16.0 Hz, 1H), 4.19 (t, *J* = 6.0 Hz, 2H), 3.86 (s, 6H), 3.85 (s, 3H), 1.75–1.59 (m, 2H), 1.52–1.32 (m, 2H), 0.94 (t, *J* = 8,0 Hz, 3H), ^13^C NMR (50 MHz, CDCl_3_) δ_C_ 167.1, 153.5, 144.6, 140.1, 130.0, 117.6, 105.2, 64.5, 61.0, 56.2, 30.9, 19.3, 13.8, IR ν_max_ (KBr, cm^−1^) 2990, 2965, 2943, 2841, 1697, 1638, 1005, 1582, 1506, 845 [54].

#### 4.1.5. (*E*)-2-Methoxyethyl-3-(3,4,5-trimethoxyphenyl)acrylate (**8**)

Yield 97%, brown oil, MM: 296,13 g/mol, m.p.: 60–62 °C; ^1^H NMR (200 MHz, CDCl_3_) δ_H_ 7.61 (d, *J* = 16.0 Hz, 1H), 6.74 (s, 2H), 6.39 (d, *J* = 16.0 Hz, 1H), 4.35 (d, *J* = 4.0 Hz, 2H), 3.86 (s, 9H), 3.65 (t, *J* = 4.0 Hz, 2H), 3.41 (s, 3H), ^13^C NMR (50 MHz, CDCl_3_) δ_C_ 167.0, 153.5, 145.2, 140.1, 129.9, 117.1, 105.3, 70.7, 63.6, 61.0, 59.2, 56.2, IR ν_max_ (KBr, cm^−1^) 3019, 2963, 2939, 2841, 1709, 1638, 1037, 1001, 1584, 1504, LS-MALDI TOF/TOF *m*/*z* [M]^+^ 296.1244 (calcd. for C_15_H_20_O_6_, 296.1260).

#### 4.1.6. (*E*)-Isopropyl 3-(3,4,5-Trimethoxyphenyl)acrylate (**9**)

Yield 96%, brown oil, MM: 280.13 g/mol; ^1^H NMR (200 MHz, CDCl_3_) δ_H_ 7.54 (d, *J* = 16.0 Hz, 1H), 6.71 (s, 1H), 6.29 (d, *J* = 16.0 Hz, 1H), 5.09 (hept, *J* = 6.00, 1H), 3.83 (s, 9H), 1.29–1.19 (m, 6H), ^13^C NMR (50 MHz, CDCl_3_) δ_C_ 166.5, 153.4, 144.4, 139.9, 130.0, 118.0, 105.2, 67.9, 61.0, 56.1, 22.0, IR ν_max_ (KBr, cm^−1^) 3019, 2978, 2938, 2841, 1703, 1638, 1584, 1505 [55].

### 4.2. General Procedure for Synthesis of Compounds ***6**, **7**, **10*** and ***13***

To a solution of 3,4,5-trimethoxycinnamic acid (0.1 g, 0.42 mmol) dissolved in 5.0 mL of acetone, 0.22 mL (1.68 mmol) of Et_3_N and 0.43 mmol of alkyl halide (RBr or RCl) were added and stirred and the reaction mixture was refluxed for 24 h, except for the reaction of synthesis of compound **10**, which was stirred for 48 h. After cooling at room temperature, the acetone of each reaction was removed under reduced pressure. The remaining content of those reactions were then diluted with 10 mL ethyl acetate and transferred to the separator funnel containing 10 mL of water. The products were extracted with 10 mL ethyl acetate three times and the during the organic phase when gathered, were successively washed with 5% (*w*/*v*) NaHCO_3_ and water, dried over anhydrous Na_2_SO_4_ and filtered. After the removal of ethyl acetate under vacuum, the products were isolated with silica gel 60-column chromatography (eluent: hexane-ethyl acetate) [56].

#### 4.2.1. (*E*)-Pentyl 3-(3,4,5-trimethoxyphenyl)acrylate (**6**)

Yield 66.5%, white solid, MM: 308.16 g/mol, m.p.: 105–106 °C; ^1^H NMR (200 MHz, CDCl_3_) δ_H_ 7.56 (d, *J* = 16.0 Hz, 1H), 6.72 (s, 2H), 6.32 (d, *J* = 16.0 Hz, 1H), 4.16 (t, *J* = 6.0 Hz, 2H), 3.84 (m, 9H), 1.76–1.58 (m, 2H), 1.41–1.27 (m, 4H), 0.90 (t, *J* = 6,0 Hz, 3H), ^13^C NMR (50 MHz, CDCl_3_) δ_C_ 167.1, 153.4, 144.5, 140.0, 130.0, 117.5, 105.1, 64.7, 61.0, 56.1, 28.5, 28.2, 22.4, 14.0, IR ν_max_ (KBr, cm^−1^) 2997, 2957, 2934, 2857, 1711, 1638, 1009, 1584, 1505, 825, LS-MALDI TOF/TOF *m*/*z* [M]^+^ 308.1621 (calcd. for C_17_H_24_O_5_, 308.1624).

#### 4.2.2. (*E*)-Decyl-3-(3,4,5-trimethoxyphenyl)acrylate (**7**)

Yield 64.5%, white solid, MM: 378.24 g/mol, m.p.: 37–38 °C; ^1^H NMR (200 MHz, CDCl_3_) δ_H_ 7.58 (d, *J* = 16.0 Hz, 1H), 6.74 (s, 2H), 6.34 (d, *J* = 16.0 Hz, 1H), 4.18 (t, *J* = 6,0 Hz, 2H), 3.87 (s, 9H), 1.76–1.60 (m, 2H), 1.35–1.15 (m, 14H), 0.86 (t, *J* = 6.0 Hz, 3H). ^13^C NMR (50 MHz, CDCl_3_) δ_C_ 167.1, 153.5, 144.6, 140.1, 130.1, 117.6, 105.2, 64.6. 61.1, 56.2, 32.0, 29.6, 29.4, 28.8, 26.1, 22.8, 14.2, IR ν_max_ (KBr, cm^−1^) 2995, 2953, 2926, 2853, 1713, 1636, 1007, 1582, 1504, 827, LS-MALDI TOF/TOF: *m*/*z* [M]^+^ 378.2406 (calcd. for C_22_H_34_O_5_ 378.2406).

#### 4.2.3. (*E*)-Benzhydryl 3-(3,4,5-trimethoxyphenyl)acrylate (**10**)

Yield 41.6% colorless oil, MM: 404.16 g/mol ^1^H NMR (200 MHz, CDCl_3_) δ_H_ 7.67 (d, *J* = 16.0 Hz, 1H), 7.41–7.22 (m, 10H), 7.02 (s, 1H), 6.76 (s, 2H), 6.48 (d, *J* = 16.0 Hz, 1H), 3.88 (s, 9H), ^13^C NMR (50 MHz, CDCl_3_) δ_C_ 166.0, 153.5, 145.5, 140.2, 129.9, 128.6, 128.0, 127.2, 126.6, 117.3, 105.3, 77.1, 61.0, 56.2. IR ν_max_ (KBr, cm^−1^) 3028, 3001, 2938, 2837, 1709, 1636, 1001, 1582, 1504, 825, LC-MALDI TOF/TOF: *m*/*z* [M]^+^ 404.1627 (calcd. for C_25_H_24_O_5_, 404.1624).

#### 4.2.4. (*E*)-4-Methylphenethyl 3-(3,4,5-Trimethoxyphenyl)acrylate (**13**)

Yield 30.6%, white solid, MM: 356.26 g/mol, m.p.: 105–106 °C; ^1^H NMR (200 MHz, CDCl_3_) δ_H_ 7.60 (d, *J* = 16.0 Hz, 1H), 7.14 (s, 4H), 6.75 (s, 2H), 6.35 (d, *J* = 16.0 Hz, 1H), 4.41 (t, *J* = 8.0 Hz, 2H), 3.90 (s, 9H), 2.96 (t, *J* = 8.0 Hz, 2H), 2.33 (s, 3H), ^13^C NMR (50 MHz, CDCl_3_) δ_C_ 166.9, 153.4, 144.8, 140.1, 136.1, 134.8, 129.9, 129.2, 128.8, 117.3, 105.2, 65.2, 61.0, 56.1, 34.8, 21.1 IR ν_max_ (KBr, cm^−1^) 3001, 2941, 2845, 1703, 1632, 1045, 1003, 1585, 1506, 820 LC-MALDI TOF/TOF *m*/*z* [M]^+^ 356.1643 (calcd. for C_21_H_24_O_5_, 356.1624).

### 4.3. General Procedure for Synthesis of Compounds ***11*** and ***12***

To a 50 mL round-bottom flask with 0.1 g (0.42 mmol) of 3,4,5-trimethoxycinnamic acid connected to a reflux condenser was added 0.03 mL (0.42 mmol) of thionyl chloride, the reaction mixture was stirred and refluxed for 1 h and the reaction was finalized after adding 0.42 mmol of respective ROH (carvacrol or phenethyl alcohol), followed by an additional hour of stir and reflux. After cooling at room temperature, the remaining content of those reactions was then diluted with 10 mL ethyl acetate and transferred to the separator funnel containing 10 mL of water. The products were extracted with 10 mL ethyl acetate three times and the organic phase when gathered were successively washed with 5% (*w*/*v*) NaHCO_3_ and water, dried over anhydrous Na_2_SO_4_ and filtered. After the removal of ethyl acetate under vacuum, the products were isolated with silica gel 60-column chromatography (eluent: hexane-ethyl acetate) [57].

#### 4.3.1. (*E*)-Carvacryl-3-(3,4,5-trimethoxyphenyl)acrylate (**11**)

Yield 52%, White solid, MM: 370.18 g/mol, m.p.: 145–146 °C; ^1^H NMR (200 MHz, CDCl_3_) δ_H_ 7.81 (d, *J* = 16.0 Hz, 1H), 7.18 (d, *J* = 8.0 Hz, 1H), 7.04 (d, *J* = 8.0 Hz, 1H), 6.94 (s, 1H), 6.83 (s, 2H), 6.58 (d, *J* = 16.0 Hz, 1H), 3.91 (s, 9H), 2.91 (Hept, *J* = 6.0 Hz, 1H), 2.18 (s, 3H), 1.26 (s, 3H), 1.23 (s, 3H), ^13^C NMR (50 MHz, CDCl_3_) δ_C_ 165.3, 153.6, 149.4, 148.2, 146.5, 140.5, 131.0, 129.8, 127.4, 124.3, 119.9, 116.5, 105.5, 61.1, 56.3, 33.7, 24.0, 16.0, IR ν_max_ (KBr, cm^−1^) 3022, 2997, 2959, 2833, 1722, 1632, 1005, 1582, 1504, 822 [58].

#### 4.3.2. (*E*)-Phenethyl 3-(3,4,5-trimethoxyphenyl)acrylate (**12**)

Yield 83%, white solid, MM: 342.15 g/mol, m.p.: 110–112 °C; ^1^H NMR (200 MHz, CDCl_3_) δ_H_ 7.60 (d, *J* = 16.0 Hz, 1H), 7.32–7.24 (m, 5H), 6.75 (s, 2H), 6.34 (d, *J* = 16.0 Hz, 1H), 4.44 (t, *J* = 8.0 Hz, 2H), 3.88 (s, 9H), 3.03 (t, *J* = 8.0 Hz, 2H), ^13^C NMR (50 MHz, CDCl_3_) δ_C_ 167.0, 153.5, 144.9, 140.1, 137.9, 130.0, 129.0, 126.7, 117.3, 105.3, 65.1, 61.0, 56.2, 35.3, IR ν_max_ (KBr, cm^−1^) 3007, 2945, 2843, 1707, 1632, 1042, 1582, 1506, 843 [59].

### 4.4. General Synthesis of Amides

To a round-bottom flask, 0.1 g (0.42 mmol) of 3,4,5-trimethoxycinnamic was dissolved in 0.84 mL of DMF and 0.06 mL (0.42 mmol) of trimethylamine. The solution was cooled in an ice water bath and 0.42 mmol of amine were added, followed by a solution of 0.42 mmol of BOP in 0.84 mL of CH_2_Cl_2_. The mixture was stirred at 0 °C for 30 min and then at room temperature for 3 h. After of the removal of CH_2_Cl_2_ under reduced pressure, the solution was diluted with 10mL of ethyl acetate and transferred to separator funnel containing 10 mL of water. The products were extracted with 10 mL ethyl acetate three times and the organic phase when gathered were washed successively with 1 N HCl, water, 1 M NaHCO_3_ and water, dried over Na_2_SO_4_, Filtered and evaporated. The residues were purified on a silica gel 60 column chromatography (eluent: hexane-ethyl acetate) [60].

#### 4.4.1. (*E*)-*N*-Butyl-3-(3,4,5-trimethoxyphenyl)acrylamide (**14**)

Yield 80%, white solid, MM: 293.16 g/mol, m.p.: 225–226 °C; ^1^H NMR (200 MHz, CDCl_3_) δ_H_ 7.50 (d, *J* = 14.0 Hz, 1H), 6.58 (s, 2H), 6.35 (d, *J* = 16.0 Hz, 1H), 6.08 (s, 1H), 3.81 (s, 9H), 3.35 (q, *J* = 6.0 Hz, 2H), 1.60–1.23 (m, 4H), 0.90 (t, *J* = 7.1 Hz, 3H), ^13^C NMR (50 MHz, CDCl_3_) δ_C_ 166.0, 153.4, 140.6, 139.4, 130.6, 120.4, 104.9, 61.0, 56.1, 39.6, 31.8, 20.2, 13.8, IR ν_max_ (KBr, cm^−1^) 3290, 3005, 2957, 2932, 2841, 1653, 1614, 1579 [61].

#### 4.4.2. (*E*)-*N*-Benzyl-3-(3,4,5-trimethoxyphenyl)acrylamide (**15**)

Yied 99.7%, white solid, MM: 327.15 g/mol, m.p.: 180–181 °C;^1^H NMR (200 MHz, CDCl_3_) δ_H_ 7.55 (d, *J* = 16.0 Hz, 1H), 7.27 (s, 5H), 6.67 (s, 2H), 6.45 (d, *J* = 16.0 Hz, 1H), 4.51 (d, 2H), 3.83 (s, 3H), 3.78 (s, 6H), ^13^C NMR (50 MHz, CDCl_3_) δ_C_ 166.0, 153.3, 141.0, 139.3, 138.2, 130.4, 128.6, 127.7, 127.4, 120.2, 104.8, 60.9, 56.0, 43.7, IR ν_max_ (KBr, cm^−1^) 3269, 2990, 2957, 2934, 2835,1653, 1620, 1582, 1505, 820 [62].

#### 4.4.3. (*E*)-*N*-(4-Methylbenzyl)-3-(3,4,5-trimethoxyphenyl)acrylamide (**16**)

Yield 97%, white solid, MM: 341.16 g/mol, m.p.: 203–205 °C; ^1^H NMR (200 MHz, CDCl_3_) δ_H_ 7.55 (d, *J* = 16.0 Hz, 1H), 7.15 (q, *J* = 8.0 Hz, 4H), 6.68 (s, 2H), 6.37 (d, *J* = 16.0 Hz, 1H), 6.24 (m, 1H), 4.48 (d, *J* = 6.0 Hz, 2H), 2.31 (s, 3H), ^13^C NMR (50 MHz, CDCl_3_) δ_C_ 165.9, 153.4, 141.2, 139.5, 137.3, 135.2, 130.5, 129.4, 127.9, 120.1, 104.9, 61.0, 56.2, 43.7, 21.2, IR ν_max_ (KBr, cm^−1^) 3292, 3001, 2934, 2837, 1653, 1607, 1580, 1504, 831 LS-MALDI TOF/TOF *m*/*z* [M + H]^+^ 342.1679 (calcd. for C_20_H_23_NO_4_, 342.1706).

#### 4.4.4. (*E*)-*N*-(4-Methoxybenzyl)-3-(3,4,5-trimethoxyphenyl)acrylamide (**17**)

Yield 71%, White solid, MM: 357.16 g/mol, m.p.: 225–226 °C; ^1^H NMR (200 MHz, CDCl_3_) δ_H_ 7.54 (d, *J* = 16.0 Hz, 1H), 7.28–7.16 (m, 2H), 6.88–6.79 (m, 2H), 6.68 (s, 2H), 6.34 (d, *J* = 14.0 Hz, 1H), 4.46 (d, *J* = 4.0 Hz, 2H), 3.85 (s, 3H), 3.83 (s, 6H), 3.78 (s, 3H), ^13^C NMR (50 MHz, CDCl_3_) δ_C_ 165.9, 159.1, 153.4, 141.2, 139.6, 130.5, 130.3, 129.3, 120.0, 114.2, 105.0, 61.0, 56.1, 55.3, 43.4, IR ν_max_ (KBr, cm^−1^) 3318, 2990, 2957, 2934, 2837, 1653, 1618, 1581, 1508, 829 [63].

#### 4.4.5. (*E*)-*N*-(4-Chlorobenzyl)-3-(3,4,5-trimethoxyphenyl)acrylamide (**18**)

Yield 91.2%, White solid, MM: 361.11 g/mol, m.p.: 215–218 °C; ^1^H NMR (200 MHz, CDCl_3_) δ_H_ 7.56 (d, *J* = 16.0 Hz, 1H), 7.40–7.11 (m, 4H), 6.70 (s, 2H), 6.38 (d, *J* = 16.0 Hz, 2H), 4.50 (d, *J* = 6.0 Hz, 2H), 3.85 (s, 3H), 3.83 (s, 6H), ^13^C NMR (50 MHz, CDCl_3_) δ_C_ 166.0, 153.4, 141.5, 139.9, 136.9, 133.3, 130.4, 129.2, 128.9, 119.8, 105.0, 61.0, 56.1, 43.1, IR ν_max_ (KBr, cm^−1^) 3294, 3044, 2959, 2932, 2837, 1651, 1616, 1582, 1506, 825 [64].

#### 4.4.6. (*E*)-*N*,*N*-Diethyl-3-(3,4,5-trimethoxyphenyl)acrylamide (**19**)

Yield 85%, white solid, MM: 293.16 g/mol, m.p.: 197–198 °C; ^1^H NMR (200 MHz, CDCl_3_) δ_H_ 7.58 (d, *J* = 14.0 Hz, 1H), 6.67 (d, *J* = 16.0 Hz, 3H), 3.86 (s, 6H), 3.83 (s, 3H) 3.46 (q, *J* = 8.0 Hz, 4H), 1.31–1.11 (m, 6H), ^13^C NMR (50 MHz, CDCl_3_) δ_C_ 165.7, 153.0, 142.5, 139.5, 131.1, 117.0, 105.0, 61.0, 56.2, 42.4, 41.1, 15.1, 13.3, IR ν_max_ (KBr, cm^−1^) 2995, 2968, 2938, 2837, 1649, 1597, 1506, 856 [65].

### 4.5. Cell Cultures and Conditions

Human glioblastoma (GBM) cell line U87MG (American Type Culture Collection; Rockville, MD, USA) within passage number 15–20 were grown in an incubator at 37 °C in a humidified atmosphere of 5% CO_2_. They were grown in Eagle’s Minimal Essential Medium from Sigma-Aldrich (Saint Louis, MO, USA), supplemented with 10% fetal bovine serum (FBS) and 100 μg/mL of each of the following antibiotics: penicillin and streptomycin [66].

### 4.6. In Vitro Evaluation of Anticancer Activity by MTT and LDH Assays

#### 4.6.1. MTT Assay

To examine the effects of compounds on cell viability, an MTT assay was performed. U87MG cells were seeded at 1 × 10^4^ cells per well in 96-well plate and allowed to attach for 24 h. Then the cells were exposed to different concentrations of **2**–**19** (0.78, 1.56, 3.125, 6.25, 12.5, 25 and 50 μg/mL) for 48 h. All the compounds were dissolved in dimethyl sulfoxide (DMSO), and diluted in serum-free culture medium. After the incubation, a 3-(4,5-dimethylthiazol-2-yl)-2,5-diphenyltetrazolium bromide (MTT) solution was applied to cell cultures according to the manufacturer’s instructions (Cayman Chemical Company^®^, Ann Arbor, MI, USA). After 3 h of incubation, MTT-formazan crystals were dissolved in DMSO and absorbance was measured at 570 nm on a plate reader and compared with control, untreated cells. The half-maximal inhibitory concentration values (IC_50_) were determined by MTT assay performed in four replicates.

#### 4.6.2. LDH Assay

Cellular viability was assessed by the assay of LDH leakage from the cell [67]. A LDH cytotoxicity assay kit purchased from Cayman Chemical Company^®^ (Ann Arbor, MI, USA) was used following instructions provided by the manufacturer. Briefly, the cells were seeded at 10^6^ cells/well in 48-well plates, treated with compound 10 as described above for 48 h. At the end of incubation 100 μL of culture medium was collected and transferred to a fresh 48-well plate. 100 μL of reaction mixture was added to each well and incubated for 30 min at room temperature. Finally, the absorbance of the cultures was measured at 490 nm using a microplate reader [68].

### 4.7. Total Antioxidant Capacity and Total Oxidant Status Assays

The commercially available kits were purchased from (Rel Assay Diagnostics^®^, Gaziantep, Turkey) to estimate the level of TAS and TOS. As a summary, the cells were seeded at 10^6^ cells/well in 48-well plates, treated with compound **10** as described above. After 48 h incubation, plasma samples of treated and untreated cultures were transferred to a new plate, mixed with reaction solution and incubate for 10 min at room temperature. After that, the absorbance was read spectrophotometrically and the level of TAS and TOS were calculated according to the given equation.

### 4.8. Apoptosis Detection by Hoechst 33,258 Staining

The control and formulation treated cells were incubated for 48 h and were cultured in six-well cell culture plates for 48 h. after incubation, the medium was removed and the cells were fixed with 4% paraformaldehyde in phosphate buffered saline at 4 °C for 30 min. Following the washing cells three times with phosphate-buffered saline (PBS). The cells were then stained with 1 μL Hoechst 33,258 (5 μg/mL; Sigma-Aldrich, Saint Louis, MO, USA) and incubated at room temperature in the dark for 5 min. Changes in nuclear morphology were visualized under a fluorescent microscope (excitation, 350 nm; emission, 460 nm; Leica^®^ DM IL LED, Wetzlar, Germany).

### 4.9. RNA Extraction and Quantitative Reverse-Transcription Polymerase Chain Reaction Analyses

5 × 10^6^ cells were incubated with IC_50_ concentrations of compound 10 in 6-well plate in 2 mL growth medium for 48 h. Total RNA was isolated from cultured cells using PureLink^®^ RNA Mini Kit (Invitrogen, Carlsbad, MA, USA) following the instructions described by the manufacturer. For quantitative reverse-transcription polymerase chain reaction (qRT-PCR), RNA was reverse-transcribed to cDNA by using a High-Capacity cDNA Reverse Transcription kit (Applied Biosystems, Foster City, CA, USA) according to the manufacturer’s protocol. Real-time PCR analyses were performed using the resulting total cDNA on a TaqMan^®^ custom plate (Applied Biosystems) designed with 15 different genes (*EGFR, AKT1, AKT2, NFκB1, NFκB1A, NFκB2, PTEN, KRAS, PIK3CA, PIK3R1, TP53, RAF1, BRAF, DVL1, FOS*). Results were normalized to the expression of 18SRNA. qRT-PCR and data collection were performed on 7500 Fast Real-Time PCR (Applied Biosystems). Data analyses were performed using the The Applied Biosystems 7500 Fast System SDS software (Foster City, CA, USA).

### 4.10. Biosafety Evaluation

Human peripheral blood cells were cultured in order to evaluate the biosafety of **10**. To assess the cytotoxicity and genotoxicity potential of **10**, MTT, LDH, Sister chromatid exchange (SCE) and 8-hydroxy-2′-deoxyguanosine assays were assessed on cultured blood cells [69,70].

### 4.11. SCE Testing

Peripheral blood samples were incubated with **10** (50 μg/mL) at 37 °C for 72 h in culture medium (PB-MAX Karyotyping Medium, Gibco^®^, Baecelona, Spain) with 5.0 mg/mL of phytohemagglutinin (Sigma Aldrich, Steinheim, Germany). 5-Bromodeoxyuridine (BrdU) (10 μg/mL) was added to cell cultures at initiation. At 1.5 h before harvesting, colcemid (0.5 μg/mL) were added to cells. Then cells were treated with a hypotonic solution (0.075 M KCl), followed by three times fixation step in methanol/acetic acid (3:1, *v*/*v*). Cell suspensions were dropped on slides and air-dried. Three days later, the slides were stained according to the fluorescence plus Giemsa (FPG) procedure. Slides were observed under a fluorescence microscope and scores were calculated as SCEs per cell.

### 4.12. Nucleic Acid Oxidation

To detect the level of 8OHdG released into the extracellular space, in accordance with the provider’s manual, a 8-Hydroxy-2′-deoxyguanosine assay kit (Cayman chemical, Ann Arbor, MI, USA) was used and performed in accordance with the provider’s manual.

### 4.13. Statistical Analysis

All experiments with U87MGs were performed in four independent experiments and the results were expressed as the mean  ±  standard error of the mean (SEM). Statistical analysis of data obtained from cell culture experiments was performed using to the statistical program SPSS software (version 20.0, SPSS, Chicago, IL, USA). One-way analysis of variance (ANOVA) followed by Duncan’s test was used for statistical comparisons of quantitative data. A *p*-value less than 0.05 was regarded as significant.

## 5. Conclusions

The assessment of the anticancer potential of piplartine analogues (**2**–**19**) has shown that changes in trimethoxycinnamic structure may reveal novel drug candidate compounds, such as compound **10**, which executed strong cytotoxic actions against the proliferation of U87MG cells. Compound **10** had a marked concentration-dependent inhibitory effect on the viability of brain cancer cells. Furthermore, in the mechanisms of cytotoxic activity of **10**, apoptotic and oxidative processes were involved and showed good potential for altering other main molecular pathways (such as Akt and NF-κB signaling) and preventing tumor development. Moreover, 3-BTA exhibited non-genotoxic and non-cytotoxic properties on healthy human cells. The obtained results are the first showing the potential antitumor activity of the compound **10** in brain human cancer cells and giving directions for future investigations concerning a detailed assessment of pharmacological action and safe formulations.

## Figures and Tables

**Table 1:** Cytotoxic activity of the compounds **2**–**19** against proliferation of U87MG cells.

Esters		Amides	
Compound	IC_50_ (μg/mL)	Compound	IC_50_ (μg/mL)
**2**	85.129 ± 0.78	**14**	13.196 ± 0.32
**3**	100.030 ± 1.12	**15**	33.128 ± 0.41
**4**	78.393 ± 0.69	**16**	22.741 ± 0.34
**5**	57.871 ± 0.71	**17**	22.654 ± 0.39
**6**	31.666 ± 0.48	**18**	43.396 ± 0.48
**7**	34.926 ± 0.54	**19**	36.072 ± 0.44
**8**	50.077 ± 0.62		
**9**	81.433 ± 0.55		
**10**	2.579 ± 0.35		
**11**	26.701 ± 0.47		
**12**	24.786 ± 0.37		
**13**	30.336 ± 0.35		

Positive control (paclitaxel): IC_50_: 2.119 ± 0.35 μg/mL.

## Supplementary Materials

Supplementary materials are available on line.

## Abbreviations

4-OH-E2	4-Hydroxy-Estradiol
8-OH-dG	8-Hydroxy-2′-deoxyguanosine
18SRNA	18S ribosomal Ribonucleic Acid
AChE	8-OH-dG-Acetylcholinesterase
ATP	Adenosine triphosphate
AKT1	Other denomination of protein kinase B (PKB) type 1
AKT2	Other denomination of protein kinase B (PKB) type 2
ANOVA	Analysis of variance
BpV-pic	Bisperoxovanadium-pic
BRAF	B-Raf proto-Oncogene, Serine/Threonine Kinase
3-BTA	(*E*)-benzhydryl 3-(3,4,5-trimethoxyphenyl)acrylate
CT	Cycle Threshold
DMSO	Dimethyl sulfoxide
cDNA	Complementary Deoxyribonucleic Acid
DVL1	Dishevelled type 1
EGF	Epidermal Growth Factor
EGFR	Epidermal Growth Factor Receptor
FBS	Fetal Bovine Serum
FPG	Fluorescence Plus Giemsa
ER	Endoplasmic Reticulum
FOS	Finkel Osteosarcoma
GBM	Glioblastoma
HGG	High-Grade Glioma
HHV	Human Herpes virus
IC_50_	The half maximal inhibitory concentration
IGF-1	Insulin-like Growth Factor-1
KRAS	Kirsten Rat Sarcoma viral oncogene homolog
LDH	Lactate Dehydrogenase
MMC	Mitomycin C
MTT	3-(4,5-Dimethylthiazol-2-yl)-2,5-diphenyltetrazolium bromide
NC	Negative Control
NFĸB	Necrosis Factor kappa B
NFkB1	Necrosis Factor kappa B1
NFkB1A	Necrosis Factor kappa B inhibitor-α
NFkB2	Necrosis Factor kappa B2
PBS	Phosphate-buffered saline
PC	Positive control
PCR	Polymerase Chain Reaction
PHWB	Peripheral Human Whole Blood
PPL	Piplartine
PTEN	Phosphatase and Tensin Homolog
PIP3K	Phosphatidylinositol-4,5-bisphosphate 3-kinase
PIK3CA	Phosphatidylinositol-4,5-bisphosphate 3-kinase catalytic
PIK3R1	Phosphatidylinositol-3-kinase regulatory subunit 1
PRDX4	Peroxiredoxin 4
RAF1	Rapidly Accelerated Fibrosarcoma type 1
ROS	Reactive Oxygen Species
RT	Radiotherapy
RT-PCR	Real Time-Polymerase Chain Reaction
SAR	Structure Activity Relationship
SCE	Sister Chromatid Exchange
SH-SY5Y	Human Neuroblastoma cell model
SV40	Simian Vacuolating virus 40
TAC	Total Antioxidant Capacity
TAS	Total Antioxidant Status
TMZ	Temozolomide
TOS	Total Oxidant Status
TP53	Tumor Protein p53
