# Supplementary material for: Piplartine Analogues and Cytotoxic Evaluation against Glioblastoma
Source: Molecules. 2018 Jun 8;23(6):1382. doi: 10.3390/molecules23061382 (PMC6099735; doi:10.3390/molecules23061382)
Supplement: Supplementary file 1 [file molecules-23-01382-s001.pdf]

Supplementary file

Article

Flávio Rogério da Nóbrega [1], Ozlem Ozdemir [2], Sheila Cristina S. Nascimento Sousa [3], Joice Nascimento Barboza [1], Hasan Turkez [2,4] and Damião Pergentino de Sousa [1,*]

[1] Laboratory of Pharmaceutical Chemistry, Universidade Federal da Paraíba, João Pessoa, 58051-085, Brazil; frnobrega@hotmail.com (F.R.d.N.); joice_nascimentob@hotmail.com (J.N.B.)
[2] Department of Molecular Biology and Genetics, Erzurum Technical University, Erzurum, 25240, Turkey; ozlem.ozdemir@erzurum.edu.tr (O.O.); hasanturkez@gmail.com (H.T.)
[3] Programa de Pós-Graduação em Engenharia Química, Universidade Federal de Sergipe, São Cristóvão, 49100-000, Brazil; shcrisnascimento@yahoo.com.br (S.C.S.N.S.)
[4] Department of Pharmacy, "G. d'Annunzio" University of Chieti-Pescara, Via dei Vestini 31, Chieti Scalo, 66013, CH, Italy
* Correspondence: damiao_desousa@yahoo.com.br; Tel.: +55-83-3216-7347

Academic Editor: name
Received: date; Accepted: date; Published: date

Abstract: Piplartine (**1**) is an alkamide extracted from plants of the genus *Piper* and shows several pharmacological properties including antitumor activity. To improve this activity a series of analogues based on **1** have been synthesized by esterification and amidation using the 3,4,5-trimethoxycinnamic acid like starting material. In study, the moieties 3-(3,4,5-trimethoxyphenyl)acrylate and 3-(3,4,5-trimethoxyphenyl)acrylamide were maintained on esters and amides respectively. Meanwhile, functional changes were exploited and it was revealed that the presence of two aromatic rings in side-chain was important to improve the cytotoxic activity against U87MG cell line, such as the compound (*E*)-benzhydryl 3-(3,4,5-trimethoxyphenyl)acrylate (**10**), an ester that exhibited strong cytotoxicity and similar potency to paclitaxel, a positive control. Compound **10** had a marked concentration-dependent inhibitory effect on viability of U87MG cell line with apoptotic and oxidative processes, showing good potential for altering main molecular pathways, preventing tumor development. Moreover, it has strong bioavailability with non-genotoxic and non-cytotoxic properties on human blood cells. In a conclusion, the findings of the present study demonstrated that compound **10** could be a promising agent that may find applications in diseases associated with oxidative stress and as a prototype for the developing of novel drugs used in treatment of glioblastoma.

Keywords: cancer; cytotoxic activity; antitumor; phenylpropanoid; alkaloid; natural product; esters; oxidative stress; *Piper*; synthetic derivatives.

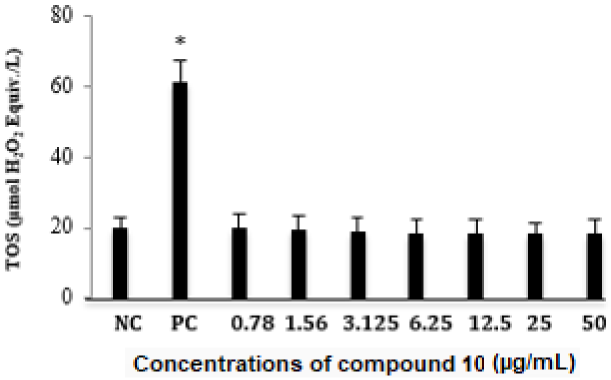

Figure 1- The effects of compound **10** on the levels of total oxidant status (TOS) in cultured U87MG cells48 h. NC: Negative control, PC: Positive control (hydrogen peroxide, 25 µM). $p < 0.05$, comparison to NC.

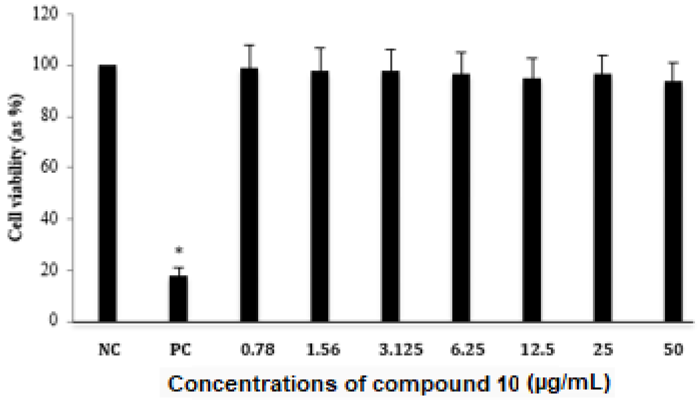

Figure 2- The *in vitro* effects of compound **10** on the cell viability of cultured human blood cells (MTT assay). Abbreviations are as in figure 2.

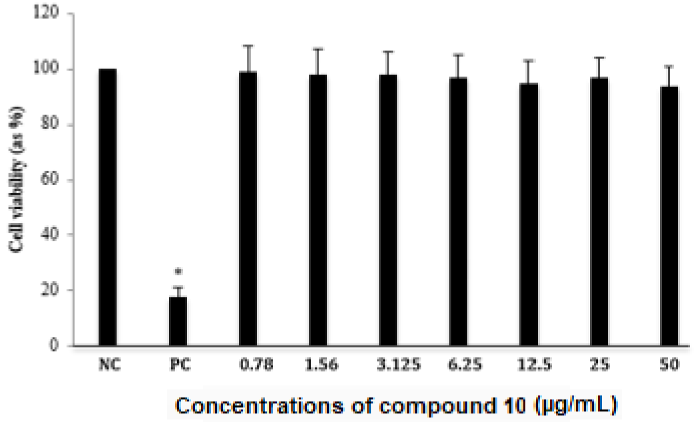

Figure 3- The *in vitro* effects of compound **10** on the cell viability of cultured human blood cells (LDH assay). Abbreviations are as in figure 2.

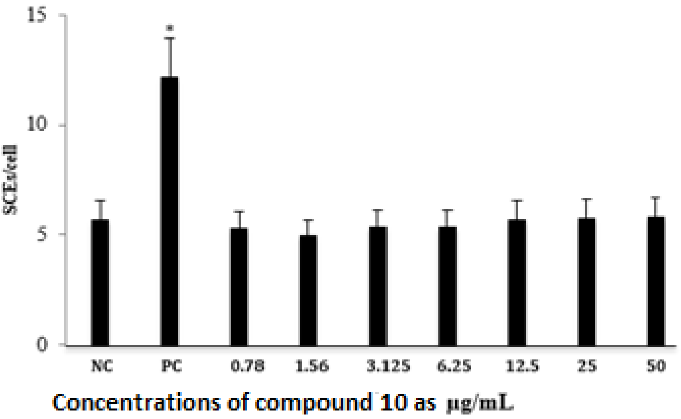

Figure 4- The *in vitro* effects of compound **10** on SCE rates in cultured human whole blood cells. NC: Negative control, PC: Positive control (Mitomycin-C, 10^{-7}M). $p < 0.05$, compared to NC.

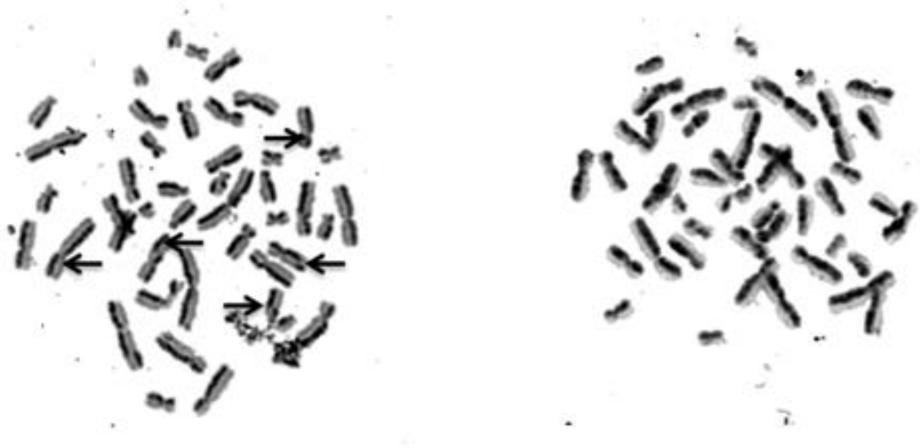

(**a**) (**b**)

Figure 5- Sample metaphases from (a) Positive control, mitomycin-c; (b) Compound **10** (50 µg/mL) treated cultures (arrows show SCE formations).

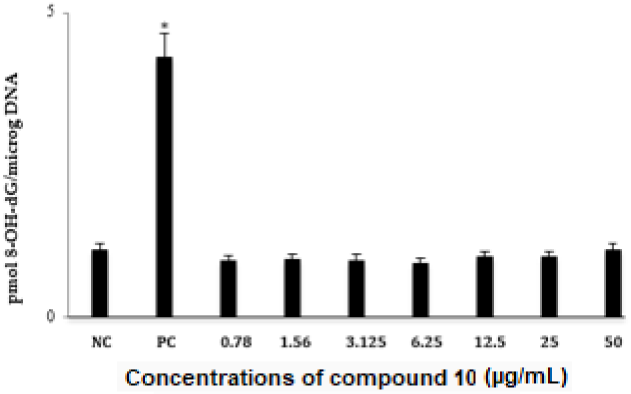

Figure 6– The levels of 8-oxo-2-deoxyguanosine (8-OH-dG) adducts in cultured human blood cells maintained 72 h in the presence of different concentrations of compound **10**.
